# Preterm Birth: A Primary Etiological Factor for Delayed Oral Growth and Development

**DOI:** 10.5005/jp-journals-10005-1316

**Published:** 2015-09-11

**Authors:** Iram Zaidi, Muhamad Nishad Thayath, Shikha Singh, Anju Sinha

**Affiliations:** Reader, Department of Pediatric and Preventive Dentistry, SBB Dental College, Ghaziabad, Uttar Pradesh, India; Professor and Head, Department of Pediatric and Preventive Dentistry, SBB Dental College, Ghaziabad, Uttar Pradesh, India; Senior Lecturer, Department of Pediatric and Preventive Dentistry, SBB Dental College, Ghaziabad, Uttar Pradesh, India; Senior Lecturer, Department of Oral and Maxillofacial Pathology, SBB Dental College, Ghaziabad, Uttar Pradesh, India

**Keywords:** Low birth weight, Oral development, Preterm birth.

## Abstract

Preterm and low birthweight children comprise approximately 6% of all live births. It is now a well-known fact that premature children experience many oral complications associated with their preterm births. Prematurely born infants have a short prenatal development period and they are prone to many serious medical problems during the neonatal period, which may affect the development of oral tissues. Adverse perinatal factors, premature birth and exceptional early adaptation to extra-uterine life and functional activity may influence dental occlusal development and symmetry in the jaws. Thus, the goal of the present paper is to elucidate further the effect of preterm birth on the development of the dentition.

**How to cite this article:** Zaidi I, Thayath MN, Singh S, Sinha A. Preterm Birth: A Primary Etiological Factor for Delayed Oral Growth and Development. Int J Clin Pediatr Dent 2015;8(3): 215-219.

## INTRODUCTION

Premature birth is an enormous global problem that is exacting a huge toll emotionally, physically, and financially on families along with medical systems. It is now a well-known fact that premature children experience many oral complications associated with their preterm births. Prematurely born infants have a short prenatal development period that makes them prone to various neonatal complications and developmental problems. According to the World Health Organization definition, a delivery is preterm when it occurs before the 37th completed week of pregnancy.^1^

In the early years of 20th century, prematurity was defined by birth weight under 2500 gm^3^ but, in 1963, Lubchenco et al reported that birth weight is also determined by the fetal growth rate in addition to gesta-tional age. The incidence of preterm birth varies greatly between populations, being an average in the range 4 to 15%.^4^ With the changing pattern of lifestyle and increase in urbanization, the frequency of preterm children has increased, but along with that the prognosis of their survival has increased.^2^

A number of etiological factors for premature births exist, many of which are associated with maternal and fetal diseases, but often the causes remains obscure.^2^ Premature children with very low birth weight (VLBW ≤ 1500 gm) or extremely low birth weight (ELBW ≤ 1000 gm) are at greater risk for short and long-term complications like hyperbilirubinemia, perinatal asphyxia, respiratory, cardiovascular, gastrointestinal, neurological problems and nutritional deficiencies. This may also include disabilities and impediments affecting physical growth and mental development.^3^

The early and long-term effects of premature birth on the physical and psychological growth and development of the child are subjects of considerable current interest. Most studies have indicated that in early childhood the preterm children show significant delay in many areas of physical and psychological growth and development. Although ‘catch-up’ growth has been reported in later childhood, some studies have indicated that long-term delays into adolescence may occur.^5^

Like other tissues of the body, the oral structures are also affected by premature birth. All these complications have been reported to be the etiological factors behind the disturbed mineralization in primary teeth (Table 1). The present paper reviews the deleterious effect of preterm birth on oral structures and their development.

### Effect on Dental Enamel

Tooth enamel is the only hard tissue in the body that is not remodeled. As a result, all of the changes in the structure caused by insults during its development are permanently registered.

Enamel formation of primary teeth begins in 14th week of intrauterine life and continues up to the first year of postnatal life.^5^ Any alteration during the prenatal, perinatal and postnatal periods, i.e. when the enamel matrix is going through the secretion or maturation phase, can result in enamel defects. As the developing tooth germ is sensitive to a wide range of systemic disturbances, it is unable to recover the damaged enamel that often acts as a repository of information of systemic insults received during development.^6^ All these factors lead to disturbances in normal calcium metabolism.^6^

**Table 1:** **Table 1:** Effect of preterm birth on oral structures

Structural changes in the dental crowns	
• Crown dilaceration from endotracheal intubation	
Palatal distortions	
• Increase in height of the palate	
• Distortions of dental arches	
Retardation of dental growth and development	
• Delay in eruption of the primary dentition	
• Delay in growth of the permanent dentition	
Cavity/decay	
• Lesion in a pit or fissure or on a smooth tooth surface with an unmistakable cavity, undermined enamel, or a detectably softened floor or wall	
Hypoplasia	
• Quantitative alteration with located reduction in the thickness of the enamel: Pits, grooves, or larger areas of missing enamel	
Demarcated opacity	
• Quantitative alteration in the translucency of the enamel of variable degree	
• Enamel of normal thickness and intact surface with demarcation starting from the normal adjacent enamel with clear limits	
• White, cream, yellow, or brown coloring	
Cleft anomalies and palatal groove	
• Palatine cleft or groove	
Other defects	
• Oral trauma (crows with fractures, avulsion, intrusion, displacement of anterior primary teeth, alteration of tooth brownish color)	
• Skeletal bone deformity (observed clinically)	

The central hypothesis for developmental defects in preterm and low birthweight infants is altered calcium homeostasis that occurs due to systemic illness, initiating from the time of conception till the end of postnatal periods. Other local effects, such as endotracheal intubation and mechanical ventilation during the postnatal period can also contribute to the condition.^5^ Consequently, ameloblasts and odontoblasts can be affected in the pre- and postnatal periods due to various maternal and child risk factors.^7^ The clinical expression of these systemic and/or local insults during the enamel matrix formation, mineralization and maturation phases result as qualitative (opacity) or quantitative (hypoplasia) defects^8^ in enamel structure.^6^

Preterm children displayed a variety of changes in the enamel like less enamel thickness, increased roughness, pits, etc. on the surface. In the preterm children, the prenatally formed enamel is the most reduced—at a level of approximately 5 to 13 times the thickness of the enamel of full-term children, which directly reflects the shortened duration in the prenatal stage of enamel formation. The same findings have been shown by Grahnen et al,^9^ they also found decrease in enamel thickness in low-birth-weight infants when compared with full-term. The reduced enamel in preterm children is likely to have resulted from both cessation/reduction of ameloblastic activity and the reduced supply of mineral to the developing teeth. The rate of apposition of human enamel has been estimated to be 0.023 mm per day.^10^

The enamel appears rough, granular, and poorly mineralized because prematurely born infants have a substantial rate of developmental defects of enamel. They also tend to have low calcium stores and disturbed calcium metabolism, with the lowest-birth-weight children most severely affected.^11^ This might be because of the reason that in preterm children, the major part of the enamel is mineralized after birth and may thus be subjected to numerous factors which might disturb the mineralization. The postnatal enamel often had partly a zone of hypomineralized enamel that may be attributed to disturbances in calcium metabolism.^8^

These defects are usually located on the primary teeth undergoing mineralization around the time of the premature birth, i.e. the primary incisors, canines and first molars, although the second primary molars may also be involved. It was previously thought that enamel defects were limited to the primary dentition only, as the permanent teeth have not yet begun their formation at the time of the preterm birth. Other studies, however, have indicated that the effects of birth prematurity may extend into the permanent dentition as well. As the permanent teeth are thought to commence their mineralization a few months after the preterm birth, it was hypothesized that there was persistence of metabolic derangements in the VLBW children well past the neonatal period which affected mineralization of the first few permanent teeth.^7^

### Effect on Tooth Eruption

Eruption of deciduous teeth and their exfoliation followed by eruption of permanent dentition is an orderly sequential and age-specific event, and is considered as an important milestone during child’s development. Racial, ethnic, sexual and individual factors can influence eruption and are usually considered in determining the standards of normal eruption. Tooth eruption is a complex and tightly regulated process which is divided into five stages namely—pre-eruptive movements, intraosseous stage, mucosal penetration, preocclusal and postocclusal stage. The tooth eruption process is influenced by both genetic and environmental factors acting during odontogenesis. The environmental, prenatal and maternal factors, diseases, nutrition, socioeconomic status and climate, etc. may affect the timing of permanent tooth eruption. The eruption of the deciduous teeth has been thought to be more genetically determined than that of the permanent dentition.^12^

The primary teeth normally develop from mid-gestation until the end of the first year of life. This process may be disrupted in preterm infants by nutritional deficiencies, exposure to certain medications, and traumatic oral manipulations. It has been suggested that nutritional deficiencies in early postnatal life play a role in the development of defective or delayed dentition. The timing of tooth eruption in prematurely born children has been found to be delayed, although it has also been reported that maturation of both the primary and permanent dentition does not differ appreciably between pre- and full-term children.^13^ Factors thought to be related to delayed tooth eruption are short gestational period,^14,15^ low birth weight^17^ and neonatal factors, including complications of prematurity, systemic disorders, duration of oral intubation, average weight gain/day, etc.^18^

### Prevalence of Dental Defects

Changes in dental enamel are one of the most noticeable oral effects of preterm birth, and may classically present as enamel hypoplasia which is defined as a quantitative loss of enamel, or as enamel opacity, which is a qualitative change in the translucency of the enamel. These defects are usually located on the primary teeth which are undergoing mineralization around the time of the premature birth, i.e. the primary incisors, canines and first molars, although the second primary molars may also be involved.^7^

The exact mechanism and etiological factors underlying these defects are not fully understood. There are hypotheses that mineral supply deficiency could be an etiological factor. The possible pathogenesis of the dental defects associated with preterm birth may be related to direct damage to the ameloblasts as in maternal infections (rubella, cytomegalovirus). Complications of pregnancy that reduce maternal serum calcium concentrations like maternal toxemia and diabetes, hyperparathyroidism, maternal calcium and vitamin D deficiencies, are often associated with preterm birth. Similarly, neonatal hypocalcemia is often associated with traumatic delivery, cesarean section, birth asphyxia and cerebral injuries (Seow 1986).^8^

Various complications associated with prematurity predispose these infants to severe metabolic derangements and hypocalcemia, and these conditions may result in disturbed enamel formation. The problem of deranged calcium metabolism occurs to varying degrees in most premature infants, because two-thirds of the individual’s stores of calcium and phosphorus accumulate during the last trimester of pregnancy and preterm infants miss much of this mineral accretion.^19^ Systemic factors, however, such as metabolic and nutritional disturbances and infections associated with the mineral loss, could cause alterations. These defects are usually located on the primary teeth undergoing mineralization around the time of the premature birth, i.e. the primary incisors, canines and first molars, although the second primary molars may also be involved. It was previously thought that enamel defects were limited to the primary dentition only, as the permanent teeth have not yet begun their formation at the time of the preterm birth. Other studies, however, have indicated that the effects of birth prematurity may extend into the permanent dentition as well. As the permanent teeth are thought to commence their mineralization a few months after the preterm birth, it was hypothesized that there was persistence of metabolic derangements in the VLBW children well past the neonatal period which affected mineralization of the first few permanent teeth. Demarcated opacities in the developing permanent teeth can be a result of long-lasting/sudden insults to the enamel-forming cells ‘ameloblasts’ during the secretory phase *(in utero),* or severe disturbances during the maturation phase in the first year of life.^20,21^ Prior studies^22^ have reported that health problems, such as infections and respiratory diseases during the first few months after birth were important risk factors for demarcated opacities in the early erupting permanent teeth.^6^

### Effects on Occlusal Relationship

Both genetics and the environment influence the development of the occlusion. Various environmental factors including—disturbances in general health and growth in childhood, masticatory muscle activity, dietary factors^23^ mouth breathing, oral habits, the mother’s and child’s nutrition and health condition, and other perinatal factors, may influence the dentition during the occlusal development period and the growth of the jaws. Individual occlusal relationships have been reported to indicate a dominance of environment over genetic factors, while some combinations of occlusal traits show noticeable genetic influence.^24^

The growth and development of dentition continues from about the age of 5 weeks *in utero* until approximately 20 years postnatally. Variations exist between individuals in the onset and direction of changes, and in the total increments in arch length, breadth and circumference.^25^ The changes in maxillary and mandibular arch length are not continuous during the development of the dentition, but occur in the form of growth periods, mainly from 4 to 6 and 10 to 14 years of age. Certain patterns of mean changes in the maxillary and mandibular arch length and breadth are generally associated with eruption of the permanent incisors, canines and premolars, when the changes are greatest.^25^

The mandible is composed of different morphogenetic and functional units, among which the condyle is considered to be a growth zone that is affected by functional alterations. The influence of the functioning of the masticatory muscles has been demonstrated in animal studies, which have shown that increased masticatory function may lead to increased sutural growth and bone apposition, and reduced muscle function to a decrease in bone mineral mass.^26^ Imbalances in the mechanical forces acting on malleable tissues may result in deformations, and gravitational and positioning forces may lead to deformations and deviation of the cranial and facial bones in immature or prematurely born infants.^14^

Oral habits, such as thumb sucking, mouth breathing or tongue thrusting, and also oromuscular forces affecting the developing dentition in formative periods, are important as etiological factors for malocclusions.^27,28^ Large tonsils can reduce the space in the mouth and cause the tongue to be low and pushed forward, pressing against the teeth of the lower jaw.^29,30^ Children with a mouth breathing habit have been shown to have a greater prevalence of class III canine and molar relationships, and greater overjet than others; while tongue thrust is an infantile habit that is apt to lead to an increased prevalence of a class III canine and molar relationship and open bite.^31^

Thus, premature birth and consequential adaptation to extra- intra-uterine life has effect on dental occlusal development, and may interfere with the development of asymmetry and lacerization.

## CONCLUSION

Preterm birth is an indicator of enamel defects and is a contributing factor of delayed oral growth and development. Considering the precarious conditions and the risks that these children are subjected to in their growth process, development and adaptation to extra-uterine life, it is necessary to bring very low and extremely low birth weight concerns to the attention of health professionals, who can offer full attention to promote better quality of life.
